# Transcriptional Truncation of the Long Coding Imprinted Gene *Usp29*

**DOI:** 10.1371/journal.pone.0158004

**Published:** 2016-06-21

**Authors:** Hongzhi He, An Ye, Joomyeong Kim

**Affiliations:** Department of Biological Sciences, Louisiana State University, Baton Rouge, LA 70803, United States of America; Harvard Medical School, UNITED STATES

## Abstract

*Usp29* (Ubiquitin-specific protease 29) is a paternally expressed gene located upstream of another imprinted gene *Peg3*. In the current study, the transcription of this long coding gene spanning a 250-kb genomic distance was truncated using a knockin allele. According to the results, paternal transmission of the mutant allele resulted in reduced body and litter sizes whereas the maternal transmission caused no obvious effects. In the paternal mutant, the expression levels of *Usp29* were reduced to 14–18% level of the wild-type littermates due to the Poly-A signal included in the knockin cassette. Expression analyses further revealed an unusual female-specific up-regulation of the adjacent imprinted gene *Zfp264* in the mutant. Consistent with this, the promoter of *Zfp264* was hypomethylated only in the female mutant. Interestingly, this female-specific hypomethylation by the knockin allele was not detected in the offspring of an interspecific crossing, indicating its sensitivity to genetic background. Overall, the results suggest that the transcription of *Usp29* may be involved in DNA methylation setting of *Zfp264* promoter in a sex-specific manner.

## Introduction

*Usp29* (Ubiquitin-Specific Protease 29) is an imprinted gene located in the proximal mouse chromosome 7/human chromosome 19q13.4 [1]. This gene is located upstream of another imprinted gene *Peg3* (Paternally Expressed Gene 3). In mice, both genes share their bi-directional promoter, and the 4-kb genomic region surrounding this promoter is methylated during oogenesis and inherited as a gametic signal [2, 3]. As a consequence, both genes are expressed mainly from the paternal allele in somatic cells [1]. Also, their spatial and temporal expression patterns are very similar, particularly high levels of expression in brain [1]. *Usp29* is composed of 9 exons spreading over a 250-kb genomic region, yet the entire ORF (Open Reading Frame) is localized within the last exon [1]. The ORF of *Usp29* shows sequence similarity to ubiquitin-specific proteases, suggesting that the protein USP29 might be involved in regulating the stability of other unknown proteins. Consistent with this, recent *in vitro* studies have identified human USP29 as an enzyme removing ubiquitin and subsequently stabilizing the protein level of Claspin, a key component controlling the DNA damage checkpoint pathway [4]. This is also consistent with the observations that human USP29 might modulate the protein levels of p53 [5].

Several imprinted domains are known to have one long gene spanning several hundred kilobases in length, yet these genes tend to be expressed as non-coding RNA. Well-known such examples include *Airn*, *Kcnq1ot1*, *Snrpn* and *Ube3a-ats* [6]. It has been proposed that the transcription of these lncRNA genes may play important roles in the establishment and/or maintenance of the imprinting of a given domain. In many cases, the transcriptional truncation of these lncRNA genes usually causes changes in the mono-allelic expression and DNA methylation levels of the surrounding genes [6–8]. In the case of the Peg3 domain, *Usp29* is regarded as a long gene given its size, 250 kb in length, although it still codes for a protein. In terms of evolutionary history, however, *Usp29* appears to have evolved with very minimal levels of functional constraints, displaying low levels of conservation in its amino acid sequence [1]. In fact, *USP29* has lost its ORF due to recent non-sense mutations in the bovine lineage and also in one ethnic group of humans (http://www.1000genomes.org/home), hinting at the possibility that *USP29* might be already in the process of becoming an lncRNA gene in these lineages of mammals [9, 10]. Given these observations, we hypothesize that the transcription itself or long transcript of *Usp29* may play some roles in the imprinting regulation of the Peg3 domain as seen in the other imprinted domains.

To test this possibility, we have generated a knockin allele truncating the transcription of mouse *Usp29* in the current study. According to the results, the truncation indeed resulted in a dramatic down-regulation of *Usp29* and also concurrent up-regulation of an adjacent imprinted gene *Zfp264*. Interestingly, the truncation also resulted in female-specific DNA hypomethylation on the promoter of *Zfp264*, suggesting that the transcription of *Usp29* may be involved in the establishment and/or maintenance of DNA methylation of *Zfp264* in a gender-specific manner.

## Results

### Generation of a knockin allele truncating the transcription of mouse *Usp29*

**Fig 1:**
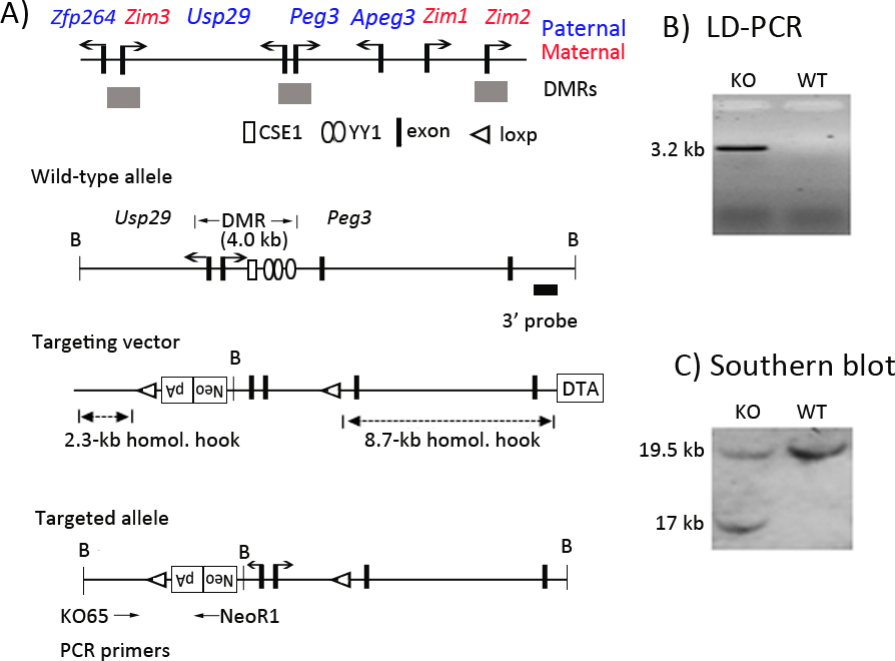
Peg3 domain and the targeting scheme. (**A**) Schematic representations of the Peg3 domain (upper panel). Each imprinted gene is indicated with an arrow. The genes with blue are paternally expressed whereas the genes with red are maternally expressed. The three DMRs are indicated with gray boxes. Targeting scheme (lower panel). The 4.0-kb Peg3-DMR contains the first exons of *Peg3* and *Usp29* and two evolutionarily conserved elements (CSE1 and CSE2 or YY1-binding site). The transcriptional direction of *Peg3* and *Usp29* is indicated with arrows, and exons are indicated with thick vertical lines. The region corresponding to the neomycin resistance gene (*NeoR*) along with the two flanking loxP sites within the targeting vector are also indicated by an open box and triangles, respectively. Arrows underneath ‘Targeted allele’ indicate primers with relative positions that were used for long-distance PCR. (**B**) LD (Long Distance)-PCR. A set of primers, KO65 and NeoR1, successfully amplified the 5.2-kb genomic fragment from the genomic DNA of a heterozygote animal (KO), but not from that of a wild-type littermate (WT), confirming the proper targeting of the KO construct. (**C**) Southern blot analyses on the DNA isolated from ES cells that had been transfected with the targeting vector. *BamH*I-digested DNA was hybridized with the 3′-side probe, showing an additional 17-kb fragment in a targeted clone.

**Fig 2:**
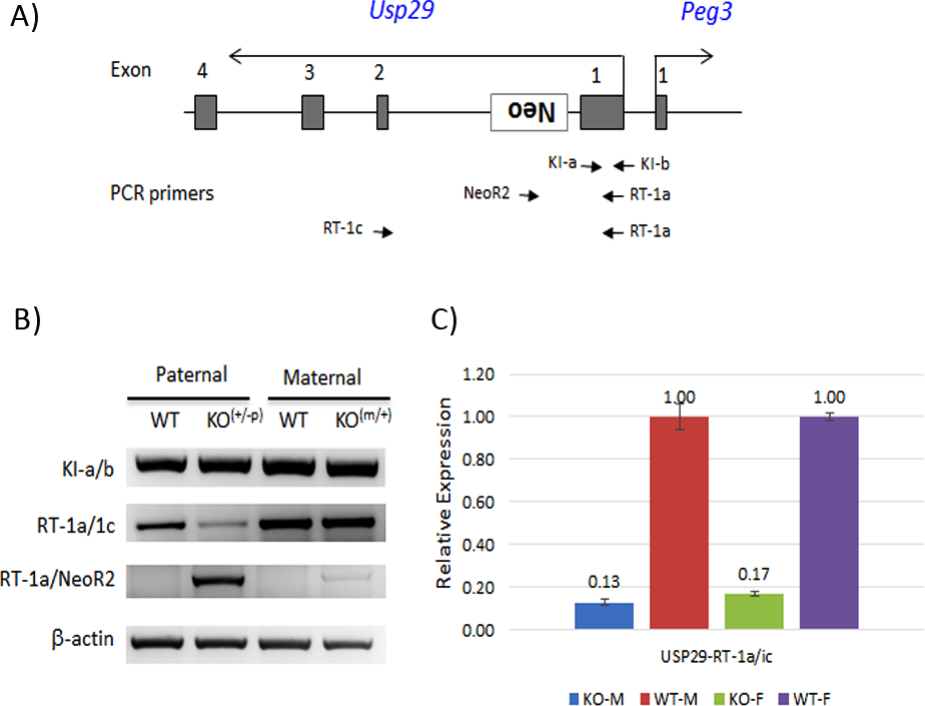
RT-PCR analyses testing the transcriptional truncation of *Usp29* by the knockin allele. (**A**) The schematic diagram indicates the positions of three primer sets (arrows) relative to the exons of *Usp29*. (**B**) A series of RT-PCR analyses were performed using the total RNA isolated from the brains of one-day-old pups with the following genotypes: wild-types (WT) and heterozygotes (KO) with the paternal (+/p) or maternal (m/+) transmission of the knockin allele. The amounts of cDNA were normalized with an internal control (β-actin). (**C**) The expression levels of *Usp29* were measured through qRT-PCR using the primer set (RT-1a/c). This series of analyses used a set of four samples with the paternal transmission of the knockin allele: WT and KO of both genders (male and female). The expression level of each sample was first normalized with an internal control, and later the normalized levels of the KO samples were compared to those of the WT samples. These relative values were presented with standard errors (S.E.).

The 4-kb genomic region harboring the bi-directional promoter of *Usp29*/*Peg3*, termed the Peg3-DMR (Differentially Methylated Region), was previously targeted to test its predicted role in establishing the maternal allele-specific DNA methylation pattern (**Fig 1)**[11]. As an ongoing effort, the current study has targeted again the same genomic region with a different scheme. In this new scheme, the 4-kb genomic region is flanked by two LoxP sites, thus allowing conditional deletion of the region later with the Cre recombinase. Compared to the original scheme, the *NeoR* (Neomycin Resistance) cassette has been re-localized from the 1^st^ intron of *Peg3* to the 1^st^ intron of *Usp29*, but with the same transcriptional direction as *Usp29*. The purpose of positioning *NeoR* in this manner was to block the transcription of *Usp29* with the Poly-A signal that had been included as part of *NeoR*. The targeting vector with this new scheme was constructed, and subsequently transfected into the ES cells of 129/SvJ (**Fig 1A**). Transfected ES cells were subsequently screened with long-distance PCR and southern blotting, identifying 20 targeted clones out of 300 ES cells (**Fig 1B and 1C**). Two independent clones with the proper targeting were injected into the blastocysts of C57BL/6J, subsequently generating 16 chimeras. Among these chimeras, two were able to generate F1 pups with the germline transmission of the targeted allele. A series of RT-PCR analyses were performed to test potential truncation of *Usp29* by the knockin allele (**Fig 2**). The primer set detecting the 1^st^ exon of *Usp29* (RT-1a/b) before the *NeoR* cassette did not show any major difference in the expression levels between the wild-type littermates and the heterozygotes inheriting the knockin allele paternally (**Fig 2B**). In contrast, the primer set flanking the *NeoR* cassette (RT-1a/1c) showed a dramatic down-regulation of *Usp29* in the heterozygotes of both genders (**Fig 2B and 2C**). As expected, a fusion transcript between exon 1 and *NeoR* (RT1a/NeoR2) was also detected by the primer set (RT-1a/NeoR2) in the heterozygotes inheriting the knockin allele paternally. Thus, the inserted *NeoR* cassette appeared to be transcribed along with the exon 1 of *Usp29*, but the transcription of this fusion transcript was truncated before the exon 2 of *Usp29*, most likely due to the Poly-A signal. Thus, this series of RT-PCR analyses confirmed the transcriptional truncation of *Usp29* by the knockin allele. Overall, these results confirmed the successful generation of the knockin allele and its subsequent transcriptional truncation of *Usp29*.

### Phenotypic effects of the knockin allele at the organism level

**Fig 3:**
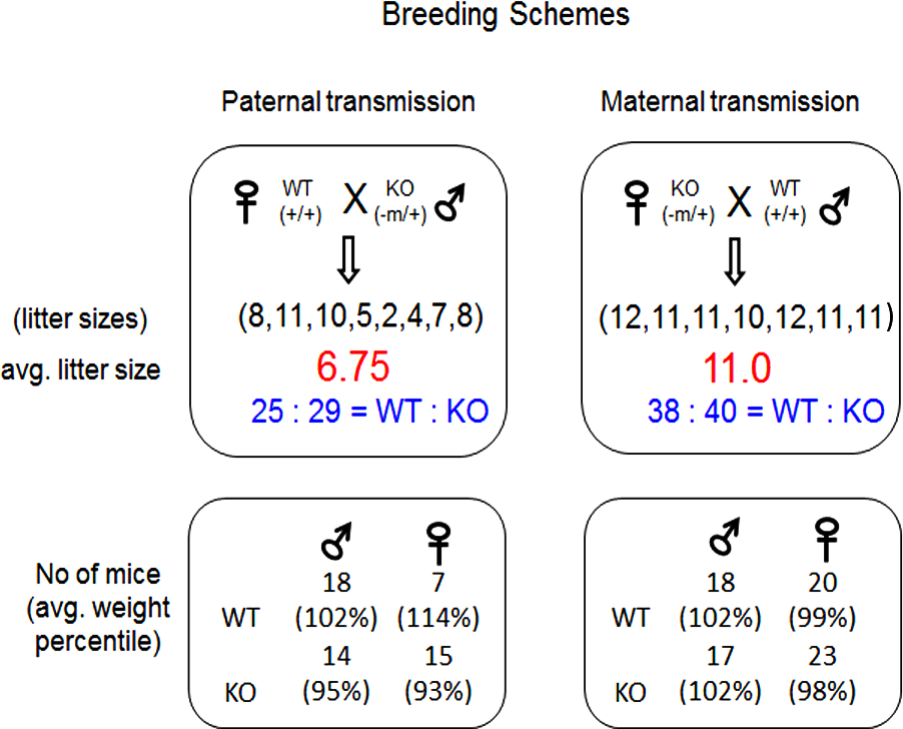
Mutational effects on the litter size and average body weight of the mouse. (top) Male and female heterozygotes inheriting the mutant allele maternally (-m/+) were bred with the wild-type littermates for breeding experiments. The average litter sizes and the transmission ratios of the knockin allele (WT versus KO) were summarized and presented for these two sets of breeding experiments. The numbers inside parentheses indicate the sizes of individual litters. (bottom) The average body weight in a percentile scale was also presented for each category of the pups that had been derived from the two sets of breeding experiments.

**Fig 4:**
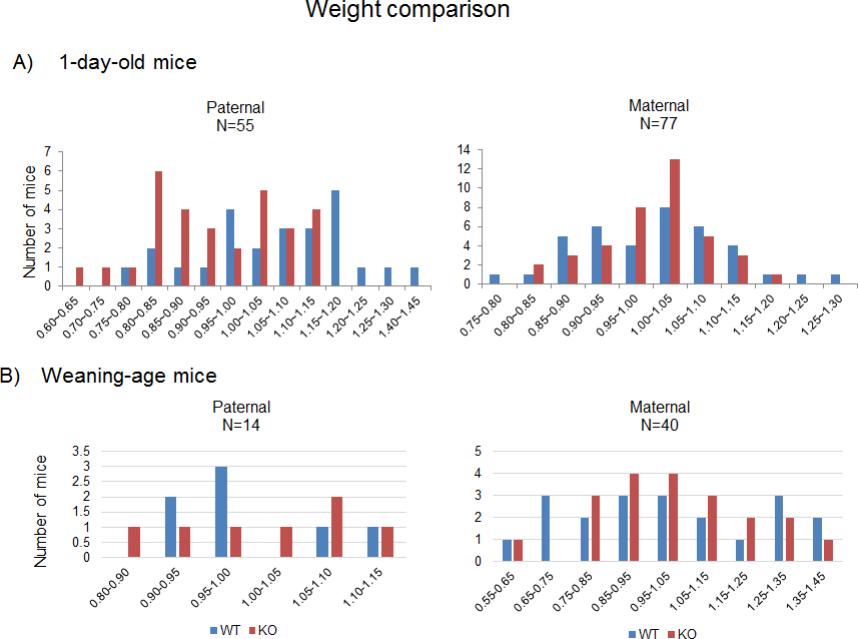
Mutational effects on the body weight of the mouse. Male and female heterozygotes were individually bred with their wild-type littermates for the paternal and maternal transmission of the knockin allele. The body weight of each pup was first divided by the average weight of a given litter, providing a percentile score for each pup. The values on the X axis indicate these percentile values, while the values on the Y axis indicate the number of mice. (**A**) The one-day-old pups inheriting the knockin allele paternally showed much smaller body weights than their littermates. (**B**) The weight profiles of the pups at the weaning age did not show any major changes between the heterozygotes and wild-type littermates in both paternal and maternal transmission of the knockin allele.

The mutational effects of the knockin allele were first analyzed at the organism level with a series of breeding experiments (**Figs 3** and **4**). Male and female heterozygotes were bred individually with the female and male wild-type littermates, generating the pups with the paternal and maternal transmission of the mutant allele, respectively. The pups derived from these breeding schemes were subsequently analyzed in terms of their gender, genotype and body weight. The results from these breeding experiments provided the following conclusions. First, the average litter size of the pups with paternal transmission of the knockin allele was smaller than the normal litter size of the mice with 129/B6-mixed background (6.75 vs 10), yet the ratio between the wild-type and the heterozygote for the mutant allele was close to the mendelian ratio (WT:KO = 25:29). This indicates no embryonic lethality associated with this mutation. However, this also suggests potential effects of the mutation on sperm and/or spermatogenesis since the average litter size was smaller than normal. In contrast, the average litter size of the pups with the maternal transmission is normal (11), and also the ratio between the wild-type and the heterozygote is close to the mendelian ratio (WT:KO = 38:40). Thus, this indicates no obvious phenotypic effects of the mutation on the survival of the pups during early embryonic stage. Second, the average body weight of the one-day-old pups with paternal transmission of the knockin allele was smaller than that of their wild-type littermates: 95 vs 102% for male (student t-test, *p*-value = 0.046545) and 93 vs 114% for female (student t-test, *p*-value = 0.002014). This becomes more obvious in the weight profile of the entire one-day-old pups used for this analysis (**Fig 4**). The heterozygotes for the knockin allele showed the peak at the 80–90% range whereas the wild-type pups showed the peak at the 110–120% range, showing significantly different weight profiles between the two groups. In contrast, the maternal transmission of the knockin allele did not cause any difference between the wild-types and the heterozygotes, displaying almost identical average body weights and also weight profiles among male and female pups. This indicates that the knockin allele has no mutational effects on body weight when inherited as the maternal allele. Third, the average body weights of the pups at the weaning age did not have a major difference between the two groups regardless of its inheritance mode, paternal or maternal (**Fig 4**). This suggests that the mutational effect of the knockin allele on body weight may be minimal during the postnatal stage. Overall, this series of breeding experiments concluded that the knockin allele causes reduced body weight and litter sizes when inherited as the paternal allele.

### Mutational effects of the knockin allele on transcriptional levels

**Fig 5:**
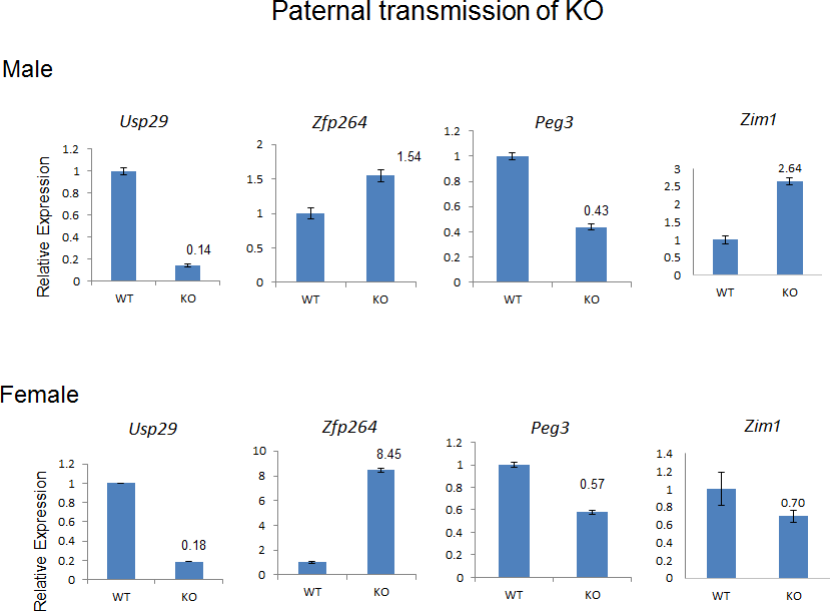
Mutational effects on transcriptional levels. A series of qRT-PCR were performed to test the mutational effects of the knockin allele on the transcriptional levels of the imprinted genes. The series of analyses used the total RNA isolated from the brain of the one-day-old pups carrying the knockin allele paternally. The expression levels of each gene were first compared between the wild-types and the heterozygotes, and the relative levels are presented in a graph with standard errors (S.E.). The expression levels of two genes, *Usp29* and *Zfp264*, were affected the most by the knockin allele.

**Fig 6:**
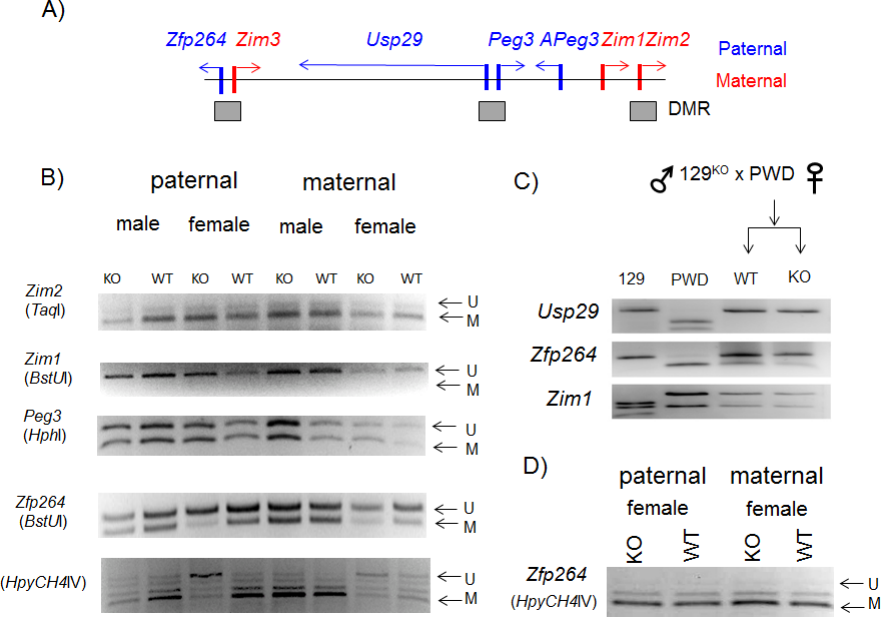
Mutational effects on DNA methylation levels. (**A**) Schematic representation of the Peg3 domain with three DMR (Differentially Methylated Regions). The genes with blue and red indicate paternally and maternally expressed genes, respectively. The DMRs are indicated with grey boxes. (**B**) A series of DNA methylation analyses were performed using the DNA isolated from two sets of the mice: one set inheriting the knockin allele paternally (lane 1–4) whereas the other set inheriting maternally (lane 5–8). Each set has two heterozygotes with both sexes. The DNA isolated from either tail or brain was treated with bisulfite conversion protocol, and subsequently used for the amplification of each target region. The amplified PCR product was analyzed with COBRA. The restriction enzyme used for each set of PCR products are shown underneath of the gene’s name. The undigested and digested DNA fragments by a given restriction enzyme are also indicated by U and M with arrows, respectively. The DNA methylation levels of the Peg3 domain were not affected by the knockin allele except the hypomethylation observed in the promoter of *Zfp264* in the female heterozygotes (lane 3, 7). (**C**) RT-PCR-based imprinting test of the genes within the Peg3 domain. This series of imprinting tests used the total RNA isolated from the brain of the F1 hybrid of the female set that had been prepared through the crossing of the heterozygotes with 129/B6 background and breeding partners with PWD/PhJ background. The product from RT-PCR was digested with a given restriction enzyme to differentiate parental alleles, which are shown in different-size DNA fragments on gel images. The imprinting status of three genes did now show any difference between WT and KO mice. (**D**) COBRA analyses were also performed using the DNA isolated from the female set of WT and KO with the paternal and maternal transmission of the knockin allele. The DNA methylation levels of the promoter of *Zfp264* were not affected in this set of F1 hybrid mice.

Next, we analyzed the mutational effects of the knockin allele on the transcriptional levels of the genes in the Peg3 domain in the following manner. Total RNA was isolated from the brain of the two sets of one-day-old pups. The first set includes the wild-types and heterozygotes of both genders with paternal transmission of the knockin allele whereas the second set includes the same combination of pups but with maternal transmission of the knockin allele. This set of RNA was used for performing qRT-PCR with several sets of primers targeting the imprinted genes in the Peg3 domain. According to the results, two genes (*Usp29* and *Zfp264*) showed the most changes in their expression levels compared to those of the wild-type littermates (**Fig 5**). The expression levels of *Usp29* were down-regulated in male (14%) and in female (18%) heterozygotes. This down-regulation was observed only in the pups with the paternal transmission, but not in the pups with the maternal transmission. This is consistent with the observation that the paternal-specific expression of *Usp29* is still maintained among the mutant animals carrying the knockin allele (**Fig 6**). This dramatic down-regulation of *Usp29* has been expected given the original purpose of the knockin allele, truncating the transcription of *Usp29* (**Fig 1**). The second most affected gene by the knockin allele turned out to be *Zfp264* in the female heterozygotes, showing a 8.4-fold increase in the expression level compared to that of the wild-type littermate. Interestingly, a similar level of up-regulation was not observed in the male heterozygotes although they showed 1.5-fold increase compared to the wild-type littermates. It is, however, somewhat difficult to recognize the significance of this level of up and down-regulation, ranging from 1.5 to 0.5-fold, mainly because of its variability between individual samples. This might also be the case for *Peg3*, in which the male and female heterozygotes showed 43 and 57% levels of the wild-type littermates, respectively. The results from the pups with the maternal transmission also showed an overall similar range of up- and down-regulations in the gene set without any dramatic change (data not shown), confirming again that the transcriptional truncation by the knockin allele is paternal allele-specific. Overall, this series of expression analyses concluded that the paternal transmission of the knockin allele results in a dramatic down-regulation of *Usp29* and a concurrent up-regulation of the adjacent gene *Zfp264* but only in females.

### Mutational effects of the knockin allele on DNA methylation levels

**Fig 7:**
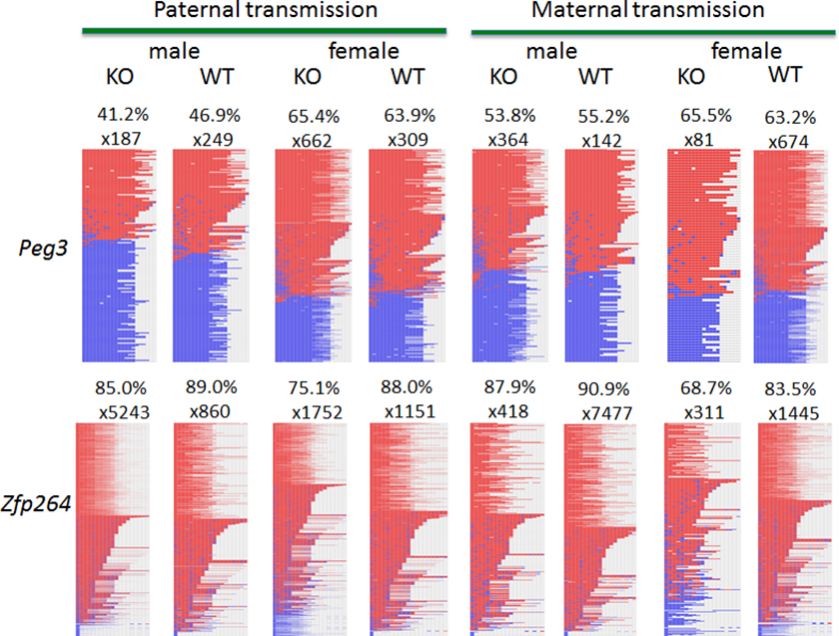
DNA methylation level analyses through individual sequencing. The DNA methylation levels of the Peg3-DMR and Zfp264 promoter were also analyzed using an NGS-based bisulfite sequencing protocol. This methylation survey used the exact same sets of PCR products that had been used for COBRA in **Fig 5B**. Thus, the results of these analyses were presented in a similar format. The two sets of 8 PCR products representing the Peg3-DMR (upper) and Zfp264 promoter (bottom) were derived from the mice with both genders that inherited the knockin allele paternally and maternally. The numbers of raw sequence reads used for calculating the overall DNA methylation level for a given locus are shown above each image. The image composed of a large number of blue and red boxes represents the graphic summary of DNA methylation patterns. Each horizontal line represents one sequence read while each small box within this line indicates one CpG dinucleotide. The blue and red boxes indicate the unmethylated and methylated CpG sites, respectively.

Potential effects of the knockin allele on the DNA methylation levels of the genes in the Peg3 domain were also tested using the DNA isolated from the same two sets of the pups used for the expression analyses (**Fig 6**). For this series of analyses, the isolated DNA was first treated with the bisulfite conversion protocol [12], which was later used as templates for PCR to amplify several target regions within the Peg3 domain. Four promoter regions were targeted for this series of DNA methylation analyses (**Fig 6A**). The amplified products from the bisulfite-converted DNA were analyzed first with COBRA (COmbined Bisulfite and Restriction Analysis)[13] (**Fig 6B**). According to the results, the knockin allele did not cause any change in the DNA methylation levels of the surveyed regions except the bi-directional promoter of *Zfp264*/*Zim3*. The DNA methylation levels of this bi-directional promoter became hypomethylated in the DNA isolated from the female heterozygotes (lane 3 in **Fig 6B**). This hypomethylation was observed repeatedly in three additional sets of female mice and also in the DNA isolated from different tissues, including brain and tail. This hypomethylation is also sex-specific, only in the female heterozygotes. Interestingly, we also observed somewhat similar hypomethylation in the set of female pups with the maternal transmission (lane 7 in **Fig 6B**). In this case, both the wild-type and heterozygotes seemed to be hypomethylated relative to the DNA methylation level of the male pups, yet the heterozygotes seemed to show much greater levels of hypomethylation. This pattern was again observed from an independent litter of pups with the maternal transmission. This was also confirmed through individual sequencing the PCR products (**Fig 7**). A set of 8 PCR products derived from the Peg3-DMR was first sequenced using a NGS (Next Generation Sequencing)-based protocol [14]. According to the results, the Peg3-DMR showed around 50% methylation levels, ranging from 41.2 to 65.5% between individuals, without any difference between the WT and KO samples. This is consistent with the results from COBRA, indicating no major effect on the methylation levels of the Peg3-DMR by the knockin allele (**Fig 6B**). In contrast, the bidirectional promoter of *Zfp264*/*Zim3* displayed much higher levels of DNA methylation than those observed from the Peg3-DMR, ranging from 68.7 to 90.9%. Among the set of 8 samples analyzed, the two females with the KO genotype showed lower levels of DNA methylation than those from WT littermates, 75.1 vs 88.0% for the paternal transmission and 68.7 vs 83.5% for the maternal transmission. It is important to note that the differences in the DNA methylation levels observed through sequencing is smaller than those from COBRA due to some unknown bias selecting methylated DNA fragments during NGS runs. Nevertheless, this again confirmed the relative hypomethylation of the promoter of *Zfp264*/*Zim3*, which was detected through COBRA (**Fig 6B**).

We also tested potential effects of the knockin allele on the imprinting status of the genes in the Peg3 domain. For this series of analyses, we used the F1 hybrid animals derived from the crossing of the knockin mutant of 129/B6 background and the PWD/PhJ strain. The RNA isolated from the brain of one-day-old pups were subsequently used for performing RT-PCR. The PCR products for each gene were digested with an enzyme to differentiate its parental alleles (**Fig 6C**). According to the results, the paternal transmission of the knockin allele did not change the imprinting status of the tested genes, showing paternal expression of *Usp29* and *Zfp264* whereas the maternal expression of *Zim1*. This is also the case for *Peg3* and *Zim2*: the paternal expression of *Peg3* and the maternal expression of *Zim2* (**S1 File**). To test the potential effects of the maternal transmission, we also repeated the same set of imprinting tests using the tissues derived from the reciprocal crossing between male PWD/PhJ and female 129/B6 with the knockin allele. The results again indicated no changes in the imprinting status of *Usp29*, *Zim1* and *Zfp264* (data not shown). This was surprising given the DNA hypomethylation observed from the promoter of *Zfp264*/*Zim3*. Thus, we further followed up this conclusion through performing DNA methylation analyses on the promoter of *Zfp264*/*Zim3* (**Fig 6D**). According to the results, interestingly, both paternal and maternal transmission of the knockin allele did not cause any change in the DNA methylation levels of the promoter of *Zfp264*/*Zim3* in the female set. This is different from the female-specific hypomethylation observed in the mice with 129/B6 background. Thus, this may be an indication that the observed DNA hypomethylation of *Zfp264*/*Zim3* in the mutant mice is sensitive to genetic background. Consistent with this prediction, the promoter of *Zfp264*/*Zim3* has been recently identified as one of the most sensitive regions by the transient suppression of DNMT1 [15]. Collectively, this series of analyses concluded that the knockin allele coincides with the DNA hypomethylation in the bi-directional promoter of *Zfp264*/*Zim3*. Interestingly, however, this phenotype seems to be sensitive to genetic background.

## Discussion

In the current study, we truncated the transcription of the long coding gene *Usp29* using a knockin allele. According to the results, the paternal transmission of the knockin allele resulted in reduced body and litter size, but the maternal transmission did not cause any major effects. The paternal transmission of the knockin allele also caused a dramatic down-regulation of *Usp29* and an up-regulation of the adjacent gene *Zfp264* in brain but only in females. Consistent with this, the promoter of *Zfp264* was hypomethylated in the female heterozygotes. Overall, the results suggest that the transcription of *Usp29* might be involved in DNA methylation setting of the promoter of *Zfp264* in a sex-specific manner.

The transcriptional truncation of *Usp29* by the knockin allele resulted in reduced body weight and litter size in the mutant mice (**Figs 3** and **4**). These phenotypes are somewhat similar to those seen previously through the other mutant alleles targeting the Peg3 domain. For instance, the knockin allele disrupting the transcription of *Peg3* also exhibited reduced body weights and litter sizes [16, 17]. *Usp29* and *Peg3* encode two different gene products, a ubiquitin-specific protease (USP29) and a DNA-binding protein (PEG3), but the overall physiological contribution by the two proteins seem to be similar, controlling fetal growth rates. This agrees well with the general pattern that imprinted genes usually control fetal growth rates [18–20]. There are, however, some differences between the two knockin alleles. The transcriptional truncation of *Usp29* has an effect on the body weight of one-day-old pups, but this phenotypic effect slowly diminishes during the postnatal stage (**Fig 4**). By contrast, the transcriptional truncation of *Peg3* has a long lasting effect throughout the lifetime. This difference might be reflecting the different stage specificity of the functions played by the affected genes. PEG3 protein may be required for the postnatal development and growth of the animals as a DNA-binding protein in brain and other tissues. In contrast, the functional contribution by the protein USP29 might be more critical during the early embryogenesis than during the postnatal stage, thus the transcriptional truncation of *Usp29* may have a limited effect on the postnatal-stage growth of the animals. This has been also demonstrated in the bovine lineage: a genomic deletion in the *Usp29*/*Mimt1* region resulted in stillbirth in cows [21]. On the other hand, it is equally possible that the different degrees of truncation by the two knockin alleles might contribute to the different levels of phenotypic severity that had been observed between the two mutant alleles. The transcriptional truncation by the knockin allele of *Usp29* seems to be somewhat incomplete or ‘leaky’ compared to that of *Peg3* since the knockin allele of *Usp29* is still maintaining 14–18% expression levels of the wild-type littermates whereas the knockin alleles of *Peg3* have almost no expression of *Peg3* [16, 17]. In sum, the phenotypes observed from the knockin allele of *Usp29* suggest that *Usp29* may be involved in controlling fetal growth rates, which is consistent with those seen from the other imprinted genes.

**Fig 8:**
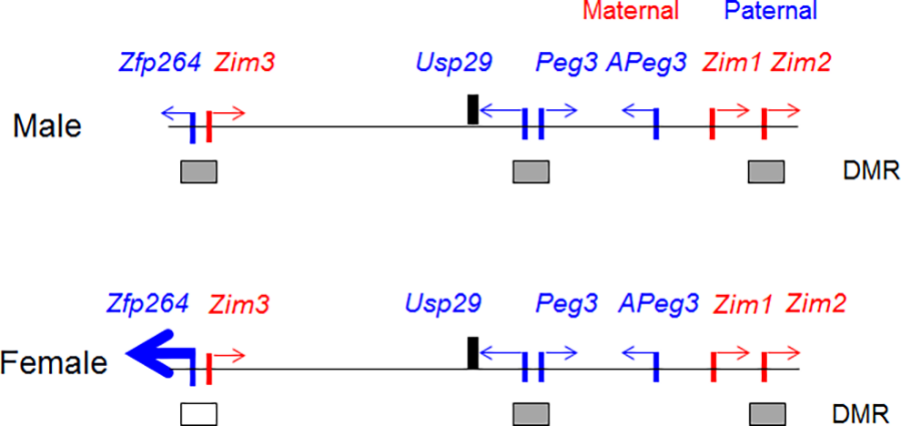
Summary of the mutational effects by the knockin allele. The mutational effects by the knockin allele are summarized in a schematic representation. The knockin allele truncates the transcription of *Usp29* in both male and female heterozygotes carrying the knockin allele paternally, indicated by the thick vertical lines. In the female heterozygotes, this transcriptional truncation of *Usp29* also results in the dramatic up-regulation of *Zfp264* in brain, indicated by a much thicker arrow compared to that in the male heterozygote. This up-regulation of *Zfp264* in the female heterozygotes coincides with the DNA hypomethylation on the promoter of *Zfp264*, indicated by the white box instead the grey box.

The knockin allele of *Usp29* has a very unusual sex-specific effect on the transcriptional and DNA methylation levels of the adjacent gene *Zfp264* (**Figs 5–7**). This phenotypic outcome has also been summarized in **Fig 8**. This outcome appears to be somewhat consistent with the observations from the other imprinted domains in that the transcriptional truncation of long genes tends to affect the transcription and DNA methylation of their adjacent genes [6, 8]. According to the results from *Airn*, a well-known lncRNA in the Igf2r imprinted domain, the transcription of this long gene by RNA polymerase II may play a role in orchestrating the long-range interaction between the cis-regulatory elements localized within the transcribed region of *Airn* and the promoters of the adjacent genes. This is also feasible in the case of *Usp29* since its transcribed region, the middle 250-kb region of the Peg3 domain, contains many cis-regulatory elements that have been identified as evolutionarily conserved regions (ECRs)[22]. The majority of these cis-regulatory elements are also marked with histone modifications, such as H3K4me1 (mono-methylation on the lysine 4 of histone 3) and/or H3K27ac (acetylation on the lysine 27 of histone 3), suggesting their potential enhancer roles for the Peg3 domain [23]. According to the results from recent studies with 3C experiments [22], one of these ECRs, ECR18, physically interacts with the promoter of several genes, *Peg3*, *Zim2* and *Zfp264*, confirming the presence of long-range interactions between these ECRs and the promoters of the imprinted genes. It is thus feasible to predict that the Pol II-driven transcription of *Usp29* may be also involved in orchestrating or forming the potential long-range interactions between some of these ECRs and the promoters of adjacent genes during early embryogenesis. If this is the case, the transcriptional truncation of *Usp29* might have some impact on establishing normal interactions, which might be, in turn, responsible for abnormal epigenetic setting, as seen in the DNA hypomethylation of *Zfp264*. Furthermore, some of these interactions could be sex-specific through testis or ovary-specific cis-regulatory elements. In that regard, it is relevant to mention that several genes in the Peg3 domain show testis-specific expression. In fact, one of the ECRs, ECR11, is known to have testis-specific histone modifications, H3K4me1 and H3K27ac [22]. Thus, some long-range interactions and subsequent epigenetic settings might be sex-specific within the Peg3 domain. As an outcome, the transcriptional truncation of *Usp29* might have different mutational outcomes between the two sexes. This might explain the sex-specific DNA hypomethylation observed from the promoter of *Zfp264* in the mutant mice. This proposed model warrants further detailed investigation in the near future; nevertheless, the observation described in the current study represents a very unique case where the transcriptional truncation of one long gene coincides with a sex-specific change in the DNA methylation levels of the promoter of the adjacent gene. Overall, this may suggest the involvement of Pol II-driven transcription in the DNA methylation setting of the Peg3 domain.

## Materials and Methods

### Ethics Statement

All the experiments related to mice were performed in accordance with National Institutes of Health guidelines for care and use of animals, and also approved by the Louisiana State University Institutional Animal Care and Use Committee (IACUC), protocol #13–061.

### Generation of a knockin allele

The targeting vector for the KO experiment was constructed with the RED/ET recombination technique (Gene Bridges)[24]. A mouse BAC (bacterial artificial chromosome) clone, RP23-178C5 (Invitrogen), was used as an initial source of DNA for isolating the 15.4-kb genomic fragment surrounding the Peg3-DMR (nucleotide positions 6,718,442–6,733,840 in the mouse chromosome 7 of mm10). The 15.4-kb fragment was isolated from the BAC clone through homologous recombination using two hooks, which were part of the two following oligonucleotides: GB primer 1 (5’-GCAAACGCCGTGTTATCAAACACCTTCATCTCAGACCACGGTCTGTGCTG-*GTCGAC-*ACAGCTTGTCTGTAAGCGGATG-3’) and GB primer 2 (5’-CAAAACAGACAACTGTGAAAAACTCACCACTCCGTTGGAGAGTTTCAAGA- *GCGGCCGC-*GCTCTCCTGAGTAGGACAAATCCG-3’). The 50 nucleotide-long sequences at the 5’-ends of both primers were the homology hooks while the sequences at the 3’-end of both primers were included for the amplification of a minimal cassette for the RED/ET recombination system (Gene Bridges, Cat. No. K002). Two restriction enzyme sites, *Sal*I and *Not*I (italicized regions), were also included as part of the sequences to be used for the linearization of the final vector and the cloning of the negative selection marker DTA (Diphtheria toxin A), respectively. PCR amplification of the minimal cassette with these two primers generated the linearized minimal cassette with two homology hooks at its 5’- and 3’-ends. The E. coli strain carrying the BAC RP23-178C5 was transformed with the expression plasmid pRedET and the linearized minimal cassette with the two homology hooks. Several colonies containing the circularized 17.8-kb vector (15.4-kb target fragment plus the 2.4-kb minimal cassette) were obtained through the ampicillin selection. The integrity of the isolated 15.4-kb genomic fragment was further confirmed through a series of restriction enzyme digestions. Two rounds of additional pRedET-based recombination were performed to insert two loxP sites that flank the 4.0-kb Peg3-DMR within the 15.4-kb interval. The positions of these loxP sites are 6,727,290–6,727,291 and 6,731,590–6,731,591 in the chromosome 7 of mm10 (**Fig 1**). While inserting the second loxP site, we intentionally left the neomycin resistance gene (*NeoR*) to truncate the transcription of *Usp29*. Finally, we subcloned the expression cassette DTA into the *Not*I site as a negative selection marker. The final 21.4-kb KO vector was linearized with *Sal*I digestion, and subsequently used for transfection into the AB2.2 ES cell line of the 129/SvJ origin (http://www.bcm.edu/dtmc/, Darwin Transgenic Mouse Core facility of Baylor College of Medicine). Transfected ES cells were first screened with a long-distance PCR scheme that can confirm the proper recombination of the 5’-side genomic fragment with the following primer set: Peg3-KO-65 (5′-TTCCTAAAGGCAAGTAGGACCT-3′) and Neo-R1 (5′-GATTCGCAGCGCATCGCCTTCT-3′). Later, a subset of the potential targeted clones identified through LD-PCR were further analyzed with southern blotting to confirm the proper recombination of the 3’-side genomic fragment. For this southern blotting, the DNA isolated from ES cells were digested with *BamH*I, and probed with the 495-bp fragment that had been amplified by PCR with the following primer set: Peg3-KO-63 (5′-ACCTTCCACTAGATTTCACCTCCT-3′) and Peg3-KO-64 (5′-CACTGCCAAAAGCATGAGATGGTC-3′). Two targeted ES cell was microinjected into the blastocysts of e3.5-embryos of the C57BL/6J (B6) mouse, producing sixteen chimeras with varying degrees of coat color contribution. Four of these chimeras were bred with 8 B6 females, finally deriving F1 mice with germline transmission of the targeted allele.

### Mouse breeding

The male and female heterozygotes carrying the knockin allele maternally were bred individually with male and female wild-type littermates. One-day-old pups derived from these breeding experiments were analyzed in terms of gender, genotype and body weight. For genotyping, genomic DNA was isolated from either clipped tails or ears by incubating the tissues overnight at 55°C in the lysis buffer (0.1 M Tris-Cl, pH 8.8, 5 mM EDTA, pH 8.0, 0.2% SDS, 0.2 M NaCl, 20 μg/ml Proteinase K). The isolated DNA was subsequently genotyped using the following primer set: BAC6331-F (5’-ATGACAAGTGGGCTTGCTGCAG-3’) and BAC6710-R (5’-GGATGTAAGATGGAGGCACTGT-3’). The genders of the pups were determined through PCR using the following primer set: mSry-F (5’-GTCCCGTGGTGAGAGGCACAAG-3’) and mSry-R (5’-GCAGCTCTACTCCAGTCTTGCC-3’). All the mice were housed at the DLAM (Division of Lab Animal Medicine) of LSU on a regular 12–12 dark-light cycle under a constant temperature 70°F and 50% humidity. All animals were given ad libitum access to water and Rodent Diet 5001. The nursing females were with Mouse Diet 5015. The mice were euthanized by CO2 asphixation in accordance with the rules and regulations set forth by the IACUC.

### RNA isolation and qRT-PCR analyses

Total RNA was isolated from the brains of one-day-old pups using a commercial kit (Trizol, Invitrogen) according to the manufacturer's protocol. The total RNA was then reverse-transcribed using the M-MLV kit (Invitrogen), and the subsequent cDNA was used as a template for PCR. The information regarding the sequences of oligonucleotides and the conditions for PCR has been published in the previous study [11].

### DNA methylation analyses

For DNA methylation analyses, genomic DNA from either tail or brain was treated with the bisulfite conversion protocol [12]. The converted DNA was then used as a template for PCR to amplify each target region. The amplified products were analyzed first with COBRA (COmbined Bisulfite and Restriction Analysis)[13], and later with individual sequencing. The information regarding the sequences of oligonucleotides and the PCR conditions for each genomic region is available through the previous study [11].

### Imprinting test

For imprinting test, the heterozygotes of the 129/B6 background were bred with the PWD/PhJ strain (Jackson Lab, Stock No. 004660). The F1 hybrid of this crossing was used for isolating total RNA and genomic DNA. The polymorphisms and restriction enzymes used for each gene’s imprinting test are also available through the previous study [11].

## Supporting Information

S1 File This file contains the additional results from the imprinting test of *Peg3* and *Zim2*, which were performed similarly as the results shown in Fig 5C. (TIF)
