# Supplementary figures and images for: Transcriptional Truncation of the Long Coding Imprinted Gene Usp29
Source: PLoS One. 2016 Jun 21;11(6):e0158004. doi: 10.1371/journal.pone.0158004 (PMC4915674; doi:10.1371/journal.pone.0158004)

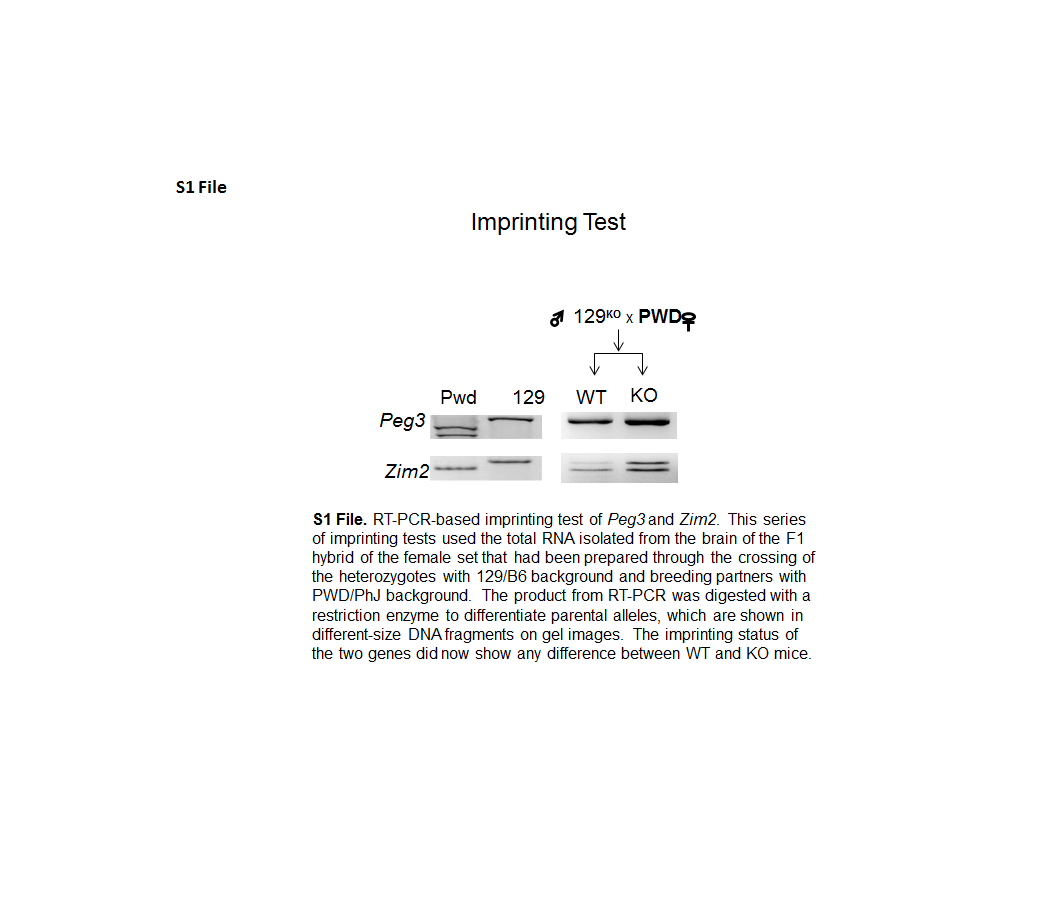

Supplement: S1 File — (TIF) [file pone.0158004.s001.tif]
